# Crystal structures of 4-(2/3-meth­oxy­phen­oxy)phthalo­nitrile

**DOI:** 10.1107/S2056989023000518

**Published:** 2023-02-17

**Authors:** Dmitry Erzunov, Anastasia Rassolova, Roman Rumyantsev, Vladimir Maizlish, Tatiana Tikhomirova, Arthur Vashurin

**Affiliations:** a Department of Inorganic Chemistry, Ivanovo State University of Chemistry and Technology, Ivanovo, Russian Federation; b Department of Fine Tune Synthesis, Ivanovo State University of Chemistry and Technology, Ivanovo, Russian Federation; c Sector of X-ray Diffraction Research, Razuvaev Institute of Metalloorganic Chemistry, Nizhnii Novgorod, Russian Federation; Universität Greifswald, Germany

**Keywords:** X-ray crystal structure, meth­oxy-phen­oxy­phthalo­nitriles, hydrogen bonds

## Abstract

The syntheses and crystal structures are reported of 4-phen­oxy-substituted phthalo­nitriles with meth­oxy groups in the *ortho*- and *meta*-positions of the terminal benzene rings, respectively, namely, 4-(2-meth­oxy­phen­oxy)phthalo­nitrile and 4-(3-meth­oxy­phen­oxy)phthalo­nitrile, both C_15_H_10_N_2_O_2_. The https://journals.iucr.org/e/issues/2023/03/00/mol­ecular structure was determined using the single-crystal X-ray diffraction method. It is shown that short contacts play a more decisive role in the mol­ecular packing compared to van der Waals inter­actions.

## Abstract 2

The syntheses and crystal structures are reported of 4-phen­oxy-substituted phthalo­nitriles with meth­oxy groups in the *ortho*- and *meta*-positions of the terminal benzene rings. Short contacts play a more decisive role in the mol­ecular packing compared to van der Waals inter­actions.

## 1. Chemical context

Phthalo­nitriles are a class of organic compounds with high thermal and oxidative stability (Laskowski *et al.*, 2016 ▸). That destruction only takes place at high temperatures facilitates using these mol­ecules as building blocks for polymer composite materials with a high degree of cross-linking (Wang *et al.*, 2019 ▸). In addition, phthalo­nitriles are among the most promising precursors for the preparation of phthalocyanine complexes of various structures based on building blocks derived from them. 
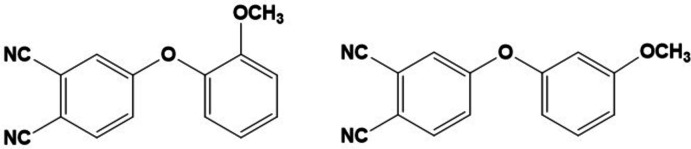


Phthalocyanines, as a result of their structural features and the possibility of introducing almost any functional moieties to their periphery, have found wide application in areas of societal and industrial importance such as catalysis, optics, medicine, light industry, etc. (Botnar *et al.*, 2020 ▸, 2021 ▸). Substituted phthalocyanines, which attract the most attention, however, are obtained from phthalo­nitriles with various fragments in the 3 and 4 positions.

Thus, it is of general inter­est to obtain functionally substituted nitriles and to study their properties. Here, we report the crystal structures of meth­oxy­phen­oxy­phthalo­nitriles with the meth­oxy group in the *meta*- and *ortho*-substitution, respectively, which have been prepared for the synthesis of the corresponding substituted phthalocyanines. X-ray diffraction data for the *ortho*-isomer are already described in the literature (Agar *et al.*, 2007 ▸). However, no discussion is provided of the influence of the structure of the substituted nitrile on the crystal-packing stabilization. The presence of oxygen atoms in the composition of the mol­ecules leads to the formation of inter­esting inter­molecular inter­actions, which are discussed in this communication.

## 2. Structural commentary

**Figure 1:**
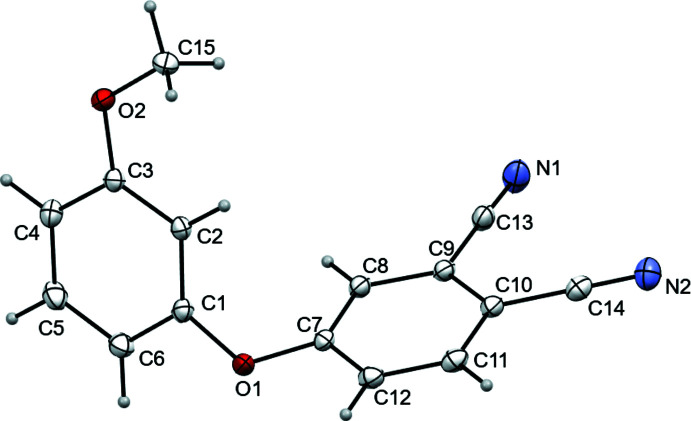
The mol­ecular structure of *o*-4-(3-meth­oxy phen­oxy)phthalo­nitrile, showing the atom labeling. Displacement ellipsoids are drawn at the 50% probability level.

Both substituted nitriles crystallize as solvent-free crystals; the structures are illustrated in Figs. 1 ▸ and 2 ▸. The phthalo­nitrile (*A*, C7–C12 atoms) and phen­oxy (*B*, C1–C6 atoms) rings are oriented at dihedral angles of 66.61 (5) and 83.84 (11)° in the cases of *meta*- and *ortho*-substitution, respectively. For both nitriles, the C13, C14, N1, N2 and O1 atoms are practically coplanar to the *A* ring with a maximum deviation of 12° in the case of the C13N1 fragment. The O1, O2, and C15 atoms and the *B* rings are essentially coplanar. The plane of the meth­oxy group (C15/O2) and its *B*-ring pivot atom (C3 or C2) is at an angle to the *B*-ring plane of only 2.21 (6)° (*meta*) or 1.43 (15)° (*ortho*). The torsion angles (C15—O2—C3—C2 for *meta* and C15—O2—C2—C1 for *ortho)* are 1.32 (15) and −179.1 (2)°, respectively.

## 3. Supra­molecular features

**Figure 3:**
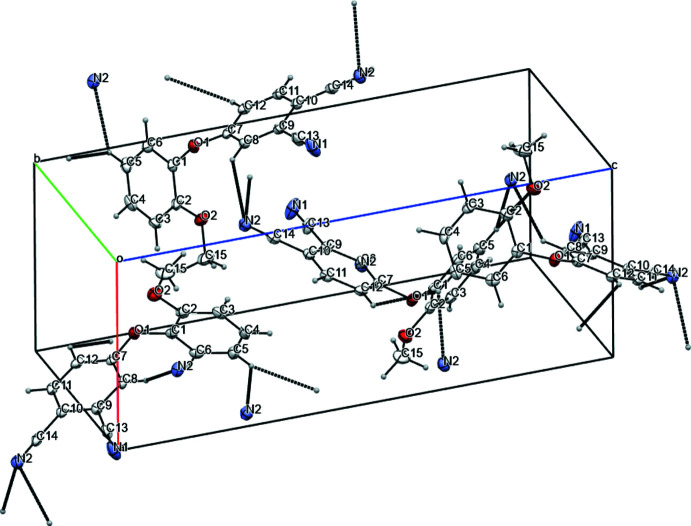
The mol­ecular structure of the *o*-4-(2-meth­oxy­phen­oxy) phthalo­nitrile dimer, linked by C–H⋯N hydrogen bonds. Displacement ellipsoids are drawn at the 50% probability level.

**Figure 4:**
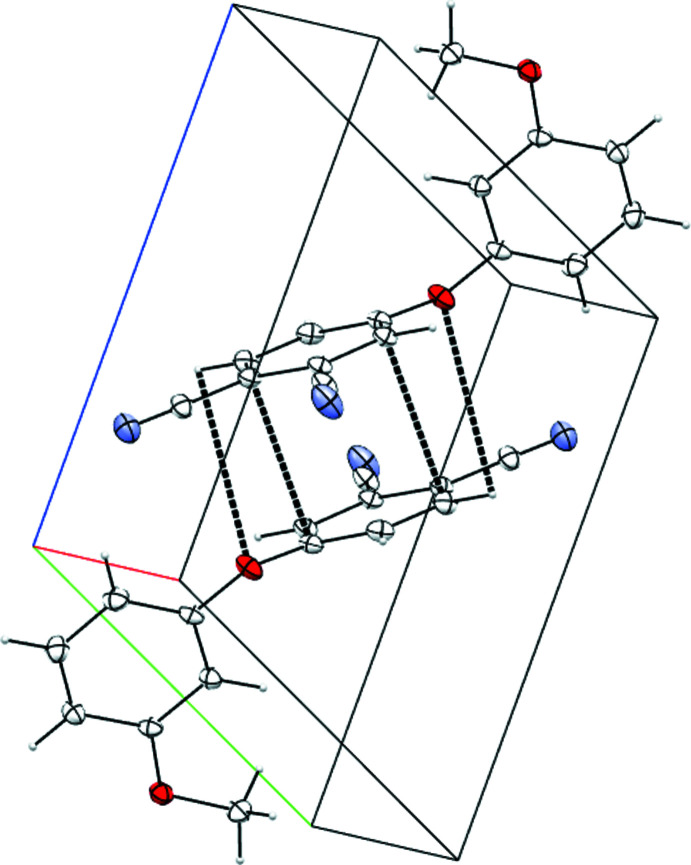
The mol­ecular structure of *m*-4-(3-meth­oxy phen­oxy)phthalo­nitrile dimer, linked by π–π-inter­actions and supporting weak H⋯O contacts. Displacement ellipsoids are drawn at the 50% probability level.

**Figure 5:**
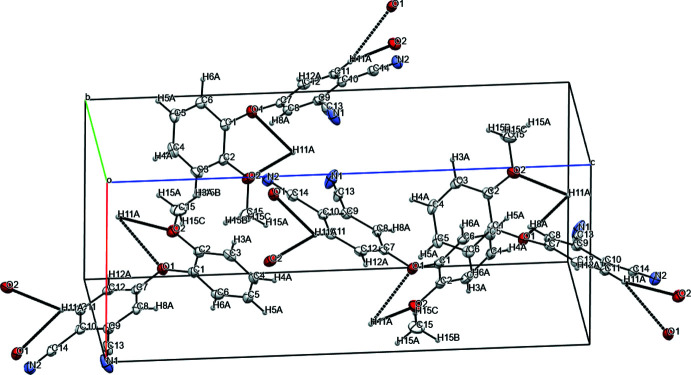
Representation of bifurcated O⋯H⋯O hydrogen bond exhibited by the *m*-4-(2-meth­oxy­phen­oxy)phthalo­nitrile dimer. Displacement ellipsoids are drawn at the 50% probability level.

**Figure 6:**
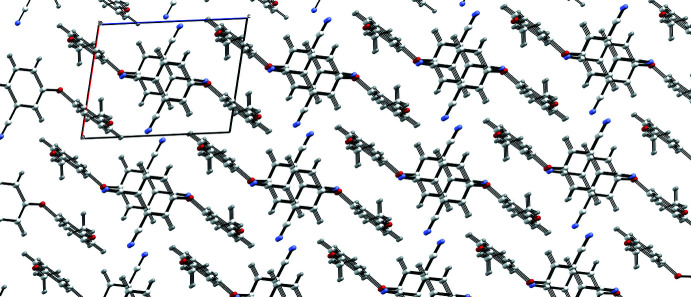
A view along the *b* axis of the crystal packing of *o*-4-(3-meth­oxy­phen­oxy)phthalo­nitrile. Inter­molecular hydrogen bonds have been removed for clarity.

In 4-(2-meth­oxy­phen­oxy)phthalo­nitrile, stabilization of the inter­molecular packing is realized mainly through the formation of hydrogen bonds between the donor C8—H8*A* group of the *A* ring with the cyano group (C14≡N2) acceptor attached to the *A* ring of an adjacent mol­ecule (C8—H8*A*⋯N2; symmetry operator: *x* + 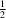 , −*y* + 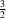 , −*z* + 1; Fig. 3 ▸, Table 1 ▸). The formation of a weaker but bifurcated inter­molecular hydrogen-bonding inter­action C11—H11⋯O1/O2(− 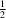 + *x*, 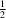 − *y*, 1 − *z*) is also found in this structure, which additionally supports the packing. In the case of 4-(3-meth­oxy­phen­oxy)phthalo­nitrile, because of the favorable spatial arrangement of two *A* rings of neighboring mol­ecules, stabilization occurs largely through respective π–π inter­actions. The planes of the *A* rings of two neighboring mol­ecules are parallel to each other, but offset (angle between the ring normal and the centroid vector is 22.6° with a slippage of 1.41 Å). The distance between the centers of the *A* rings is 3.6632 (6) Å (centroid–centroid distance). These geometric characteristics imply the presence of a significant inter­molecular π–π attraction (Janiak, 2000 ▸). The hydrogen atom of the aromatic C11—H11 moiety of one and the O1 oxygen atom of the adjacent mol­ecule may be engaged in additional bidirectional contacts (Fig. 4 ▸), which support the π–π inter­action as well as its slippage. In both cases, a number of weaker hydrogen-bonding contacts are observed, comprising additional contributions to the stabilization of the crystal structures. Thus, the packing of the *ortho*-isomer exhibits in total eight inter­molecular hydrogen-bonding inter­actions, while for the *meta*-isomer, in addition to the π–π inter­action, five hydrogen bonds are observed (Tables 1 ▸, 2 ▸, Fig. 5 ▸). The resulting crystal packings for the title 4-(2/3-meth­oxy­phen­oxy)phthalo­nitriles are shown in Figs. 6 ▸ and 7 ▸.

## 4. Database survey

A survey of the CSD (Groom *et al.*, 2016 ▸) using ConQuest version 2022 3.0 (Bruno *et al.*, 2002 ▸) for closely related 4-(phen­oxy)phthalo­nitriles with ether-functionalized substituents on the phen­oxy moiety in the *ortho*- and *meta*-positions results in only two and one hits, respectively. The *ortho*-isomers are a phthalo­nitrile dimer bridged by the *o*-phen­oxy moiety (refcode: NAGJEN; Köç *et al.*, 2016 ▸), and the same mol­ecule as the one reported here (refcode: JEVNII; Ağar & Ocak İskeleli, 2007 ▸). The *meta*-isomer is also a phthalo­nitrile dimer now bridged by the *m*-phen­oxy moiety (refcode: HAMVIB; Deveci *et al.*, 2004 ▸). Notably, with regard to the phthalo­nitrile dimers, π–π-stacking is observed for the *meta*-isomer but not for the *ortho*-isomer; the same observation was made for the two title compounds.

## 5. Synthesis and crystallization

Materials and physical methods: All reagents were purchased from Sigma–Aldrich. Reaction progress was monitored by thin-layer chromatography (TLC) on silica-gel plates.

Synthesis of substituted phthalo­nitriles: 4-nitro­phthalo­nitrile and 2/3-meth­oxy­phenol in a 1:1 molar ratio were placed in a flask and dissolved in DMF. Further, after complete dissolution of the reagents, 1 mol of potassium carbonate and 1/3 portion of water (in relation to DMF) were added to the mixture. The reaction mass was stirred at 353–363 K for 2.5 h, after which it was cooled to 278 K and poured into a threefold excess (by volume) of 15% aqueous NaCl solution. The precipitate was filtered off, recrystallized from 50% aqueous 2-propanol solution and then dried at 343 K. As a result, light crystals of 4-(2-meth­oxy­phen­oxy) phthalo­nitrile (75%) and 4-(3-meth­oxy­phen­oxy) phthalo­nitrile (89%) were obtained, respectively. Crystals were obtained by slow evaporation of solvent from a saturated solution of phthalo­nitriles in chloro­form.

## 6. Refinement

Crystal data, data collection and structure refinement details are summarized in Table 3 ▸. All hydrogen atoms were placed in calculated positions and were refined using a riding model [*U*_iso_(H) = 1.5*U*_eq_(C) for CH_3_ groups and *U*_iso_(H) = 1.2*U*_eq_(C) for other groups*)*.

## Supplementary Material

Crystal structure: contains datablock(s) o-C15H10N2O2, m-C15H10N2O2, global. DOI: 10.1107/S2056989023000518/yz2027sup1.cif

Structure factors: contains datablock(s) o-C15H10N2O2. DOI: 10.1107/S2056989023000518/yz2027o-C15H10N2O2sup3.hkl

Supporting information file. DOI: 10.1107/S2056989023000518/yz2027o-C15H10N2O2sup6.cml

Supporting information file. DOI: 10.1107/S2056989023000518/yz2027o-C15H10N2O2sup7.mol

Structure factors: contains datablock(s) m-C15H10N2O2. DOI: 10.1107/S2056989023000518/yz2027m-C15H10N2O2sup2.hkl

Supporting information file. DOI: 10.1107/S2056989023000518/yz2027m-C15H10N2O2sup6.mol

Supporting information file. DOI: 10.1107/S2056989023000518/yz2027m-C15H10N2O2sup7.cml

CCDC references: 2215782, 2215781

Additional supporting information:  crystallographic information; 3D view; checkCIF report

## Figures and Tables

**Figure 2:**
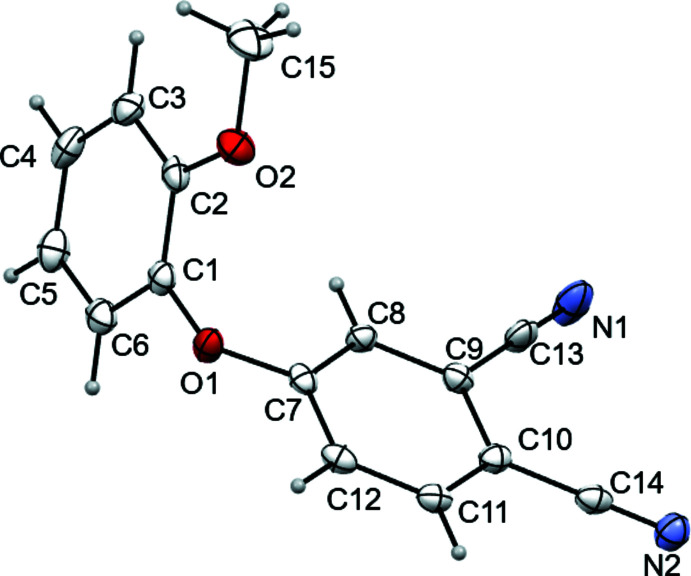
The mol­ecular structure of *m*-4-(2-meth­oxy phen­oxy)phthalo­nitrile, showing the atom labeling. Displacement ellipsoids are drawn at the 50% probability level.

**Figure 7:**
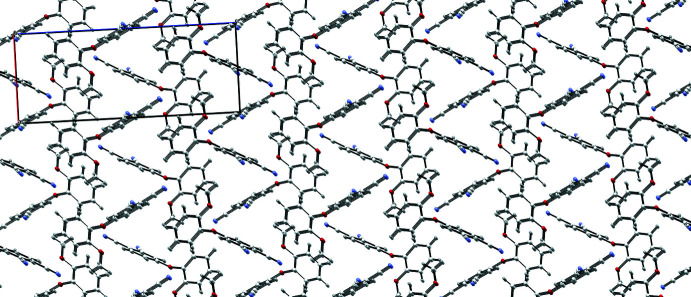
A view along the *b* axis of the crystal packing of *m*-4-(2-meth­oxy­phen­oxy)phthalo­nitrile. Inter­molecular hydrogen bonds have been removed for clarity.

**Table 1:** Hydrogen-bond geometry (Å, °) for *o*-C_15_H_10_N_2_O_2_ [Chem scheme1]

*D*—H⋯*A*	*D*—H	H⋯*A*	*D*⋯*A*	*D*—H⋯*A*
C3—H3*A*⋯O2^i^	0.95	2.95	3.629 (3)	129
C5—H5*A*⋯N2^ii^	0.95	2.66	3.394 (3)	134
C5—H5*A*⋯N1^iii^	0.95	2.90	3.557 (3)	128
C5—H5*A*⋯O1^iv^	0.95	2.96	3.453 (3)	113
C8—H8*A*⋯N2^v^	0.95	2.69	3.469 (3)	140
C11—H11*A*⋯O2^vi^	0.95	2.82	3.735 (3)	161
C11—H11*A*⋯O1^vi^	0.95	2.75	3.546 (3)	142
C12—H12*A*⋯N1^vii^	0.95	2.44	3.199 (3)	137

Symmetry codes: (i) 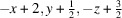 ; (ii) 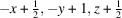 ; (iii) 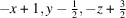 ; (iv) 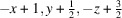 ; (v) 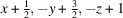 ; (vi) 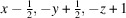 ; (vii) 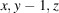 .

**Table 2:** Hydrogen-bond geometry (Å, °) for *m*-C_15_H_10_N_2_O_2_ [Chem scheme1]

*D*—H⋯*A*	*D*—H	H⋯*A*	*D*⋯*A*	*D*—H⋯*A*
C2—H2*A*⋯N2^i^	0.95	2.64	3.5839 (13)	175
C8—H8*A*⋯O2^ii^	0.95	2.47	3.3447 (11)	153
C11—H11*A*⋯N1^iii^	0.95	2.62	3.2718 (13)	126
C12—H12*A*⋯N1^iii^	0.95	2.74	3.3325 (13)	121
C12—H12*A*⋯O2^iv^	0.95	2.67	3.5343 (12)	151

Symmetry codes: (i) 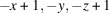 ; (ii) 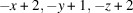 ; (iii) 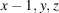 ; (iv) 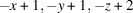 .

**Table 3:** Experimental details

	*o*-C_15_H_10_N_2_O_2_	*m*-C_15_H_10_N_2_O_2_
Crystal data		
*M*_r_	250.25	250.25
Crystal system, space group	Orthorhombic, *P*2_1_2_1_2_1_	Triclinic, *P* 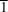
Temperature (K)	100	100
*a*, *b*, *c* (Å)	7.7329 (3), 8.2536 (3), 19.2301 (7)	8.0609 (3), 8.4672 (4), 9.9999 (4)
α, β, γ (°)	90, 90, 90	104.638 (1), 95.078 (1), 110.570 (1)
*V* (Å^3^)	1227.35 (8)	606.31 (4)
*Z*	4	2
Radiation type	Mo *K*α	Mo *K*α
μ (mm^−1^)	0.09	0.09
Crystal size (mm)	0.24 × 0.24 × 0.07	0.45 × 0.30 × 0.27

Data collection		
Diffractometer	Bruker D8 Quest (CMOS)	Bruker D8 Quest (CMOS)
Absorption correction	Multi-scan (*SADABS*; Krause *et al.*, 2015 ▸)	Multi-scan (*SADABS*; Krause *et al.*, 2015 ▸)
*T*_min_, *T*_max_	0.903, 0.971	0.903, 0.971
No. of measured, independent and observed [*I* > 2σ(*I*)] reflections	19395, 3035, 2616	10029, 3395, 3040
*R*_int_	0.046	0.017
(sin θ/λ)_max_ (Å^−1^)	0.667	0.694

Refinement		
*R*[*F* ^2^ > 2σ(*F* ^2^)], *wR*(*F* ^2^), *S*	0.045, 0.095, 1.09	0.039, 0.105, 1.06
No. of reflections	3035	3395
No. of parameters	173	173
H-atom treatment	H-atom parameters constrained	H-atom parameters constrained
Δρ_max_, Δρ_min_ (e Å^−3^)	0.21, −0.32	0.37, −0.27
Absolute structure	Flack *x* determined using 931 quotients [(*I* ^+^)−(*I* ^−^)]/[(*I* ^+^)+(*I* ^−^)] (Parsons *et al.*, 2013 ▸)	–

Computer programs: *APEX3* and *SAINT* (Bruker, 2003 ▸), *SHELXT2014/5* (Sheldrick, 2015*a* ▸), *SHELXL2018/3* (Sheldrick, 2015*b* ▸), *SHELXTL* (Sheldrick, 2008 ▸), and *Mercury* (Macrae *et al.*, 2020 ▸).

## supplementary crystallographic information

### 4-(2-Methoxyphenoxy)benzene-1,2-dicarbonitrile (o-C15H10N2O2) Crystal data

**Table:**

C_15_H_10_N_2_O_2_	*D*_x_ = 1.354 Mg m^−^^3^
*M**_r_* = 250.25	Mo *K*α radiation, λ = 0.71073 Å
Orthorhombic, *P*2_1_2_1_2_1_	Cell parameters from 9781 reflections
*a* = 7.7329 (3) Å	θ = 2.7–30.1°
*b* = 8.2536 (3) Å	µ = 0.09 mm^−^^1^
*c* = 19.2301 (7) Å	*T* = 100 K
*V* = 1227.35 (8) Å^3^	Prism, colorless
*Z* = 4	0.24 × 0.24 × 0.07 mm
*F*(000) = 520	

### 4-(2-Methoxyphenoxy)benzene-1,2-dicarbonitrile (o-C15H10N2O2) Data collection

**Table:**

Bruker D8 Quest (CMOS) diffractometer	2616 reflections with *I* > 2σ(*I*)
Radiation source: microfocus tube	*R*_int_ = 0.046
ω scans	θ_max_ = 28.3°, θ_min_ = 2.1°
Absorption correction: multi-scan (SADABS; Krause *et al.*, 2015)	*h* = −10→10
*T*_min_ = 0.903, *T*_max_ = 0.971	*k* = −11→11
19395 measured reflections	*l* = −25→25
3035 independent reflections	

### 4-(2-Methoxyphenoxy)benzene-1,2-dicarbonitrile (o-C15H10N2O2) Refinement

**Table:**

Refinement on *F*^2^	Secondary atom site location: difference Fourier map
Least-squares matrix: full	Hydrogen site location: inferred from neighbouring sites
*R*[*F*^2^ > 2σ(*F*^2^)] = 0.045	H-atom parameters constrained
*wR*(*F*^2^) = 0.095	*w* = 1/[σ^2^(*F*_o_^2^) + (0.0385*P*)^2^ + 0.3865*P*] where *P* = (*F*_o_^2^ + 2*F*_c_^2^)/3
*S* = 1.09	(Δ/σ)_max_ < 0.001
3035 reflections	Δρ_max_ = 0.21 e Å^−^^3^
173 parameters	Δρ_min_ = −0.32 e Å^−^^3^
0 restraints	Absolute structure: Flack *x* determined using 931 quotients [(*I*^+^)-(*I*^-^)]/[(*I*^+^)+(*I*^-^)] (Parsons *et al.*, 2013)
Primary atom site location: dual	

### 4-(2-Methoxyphenoxy)benzene-1,2-dicarbonitrile (o-C15H10N2O2) Special details

**Table:**

Geometry. All esds (except the esd in the dihedral angle between two l.s. planes) are estimated using the full covariance matrix. The cell esds are taken into account individually in the estimation of esds in distances, angles and torsion angles; correlations between esds in cell parameters are only used when they are defined by crystal symmetry. An approximate (isotropic) treatment of cell esds is used for estimating esds involving l.s. planes.

### 4-(2-Methoxyphenoxy)benzene-1,2-dicarbonitrile (o-C15H10N2O2) Fractional atomic coordinates and isotropic or equivalent isotropic displacement parameters (Å^2^)

**Table:**

	*x*	*y*	*z*	*U*_iso_*/*U*_eq_	
O1	0.6870 (2)	0.31404 (19)	0.64727 (8)	0.0195 (4)	
O2	0.9772 (2)	0.4854 (2)	0.66084 (9)	0.0268 (4)	
N1	0.4498 (4)	0.9100 (3)	0.50573 (12)	0.0377 (6)	
N2	0.3018 (3)	0.6112 (3)	0.36049 (11)	0.0244 (5)	
C1	0.7085 (3)	0.4120 (3)	0.70640 (12)	0.0172 (5)	
C2	0.8609 (3)	0.5003 (3)	0.71355 (12)	0.0193 (5)	
C3	0.8807 (3)	0.5958 (3)	0.77286 (13)	0.0235 (5)	
H3A	0.982511	0.658542	0.779084	0.028*	
C4	0.7515 (3)	0.5990 (3)	0.82256 (12)	0.0249 (6)	
H4A	0.765466	0.665266	0.862552	0.030*	
C5	0.6029 (3)	0.5081 (3)	0.81540 (12)	0.0242 (5)	
H5A	0.516618	0.509974	0.850546	0.029*	
C6	0.5809 (3)	0.4138 (3)	0.75631 (12)	0.0210 (5)	
H6A	0.478840	0.351196	0.750360	0.025*	
C7	0.6104 (3)	0.3826 (3)	0.59041 (11)	0.0160 (5)	
C8	0.5802 (3)	0.5471 (3)	0.58379 (11)	0.0166 (5)	
H8A	0.611760	0.619803	0.619924	0.020*	
C9	0.5027 (3)	0.6041 (3)	0.52311 (11)	0.0174 (5)	
C10	0.4544 (3)	0.4968 (3)	0.46957 (11)	0.0170 (5)	
C11	0.4861 (3)	0.3315 (3)	0.47775 (12)	0.0178 (5)	
H11A	0.454353	0.257754	0.442035	0.021*	
C12	0.5635 (3)	0.2752 (3)	0.53775 (12)	0.0180 (5)	
H12A	0.584819	0.162514	0.543185	0.022*	
C13	0.4736 (3)	0.7744 (3)	0.51398 (12)	0.0235 (5)	
C14	0.3705 (3)	0.5595 (3)	0.40833 (12)	0.0187 (5)	
C15	1.1358 (3)	0.5737 (4)	0.66790 (16)	0.0365 (7)	
H15A	1.209911	0.552231	0.627585	0.055*	
H15B	1.195310	0.539339	0.710413	0.055*	
H15C	1.110784	0.689945	0.670515	0.055*	

### 4-(2-Methoxyphenoxy)benzene-1,2-dicarbonitrile (o-C15H10N2O2) Atomic displacement parameters (Å^2^)

**Table:**

	*U*^11^	*U*^22^	*U*^33^	*U*^12^	*U*^13^	*U*^23^
O1	0.0259 (9)	0.0151 (8)	0.0175 (8)	0.0017 (7)	−0.0029 (7)	−0.0003 (6)
O2	0.0213 (8)	0.0321 (10)	0.0270 (9)	−0.0034 (8)	0.0053 (8)	0.0023 (8)
N1	0.0639 (18)	0.0183 (11)	0.0310 (12)	0.0001 (11)	−0.0236 (13)	−0.0027 (10)
N2	0.0281 (11)	0.0235 (11)	0.0216 (11)	0.0009 (10)	−0.0029 (10)	−0.0021 (9)
C1	0.0213 (11)	0.0130 (10)	0.0172 (11)	0.0032 (10)	−0.0023 (10)	0.0017 (9)
C2	0.0193 (11)	0.0204 (11)	0.0183 (11)	0.0025 (10)	0.0003 (9)	0.0067 (10)
C3	0.0216 (12)	0.0234 (13)	0.0255 (12)	−0.0002 (11)	−0.0092 (11)	0.0033 (10)
C4	0.0317 (14)	0.0266 (13)	0.0165 (12)	0.0070 (12)	−0.0083 (10)	−0.0031 (10)
C5	0.0267 (13)	0.0280 (13)	0.0177 (11)	0.0067 (12)	0.0025 (10)	0.0004 (10)
C6	0.0197 (11)	0.0193 (12)	0.0241 (12)	0.0019 (10)	−0.0016 (10)	0.0053 (10)
C7	0.0137 (10)	0.0174 (11)	0.0168 (10)	−0.0005 (9)	0.0038 (9)	0.0016 (9)
C8	0.0192 (11)	0.0162 (11)	0.0144 (10)	−0.0032 (9)	0.0003 (9)	−0.0029 (9)
C9	0.0183 (11)	0.0150 (11)	0.0187 (11)	−0.0016 (9)	0.0010 (10)	−0.0004 (9)
C10	0.0162 (10)	0.0183 (11)	0.0164 (10)	−0.0011 (10)	0.0009 (9)	−0.0026 (9)
C11	0.0162 (11)	0.0183 (11)	0.0191 (11)	−0.0029 (9)	0.0020 (9)	−0.0042 (9)
C12	0.0175 (11)	0.0123 (11)	0.0243 (12)	0.0000 (9)	0.0028 (10)	−0.0015 (9)
C13	0.0320 (14)	0.0208 (13)	0.0175 (11)	−0.0029 (11)	−0.0098 (11)	−0.0038 (10)
C14	0.0196 (11)	0.0167 (11)	0.0199 (12)	−0.0030 (9)	0.0021 (10)	−0.0054 (9)
C15	0.0195 (12)	0.0447 (17)	0.0451 (17)	−0.0050 (13)	0.0056 (12)	0.0081 (14)

### 4-(2-Methoxyphenoxy)benzene-1,2-dicarbonitrile (o-C15H10N2O2) Geometric parameters (Å, º)

**Table:**

O1—C7	1.366 (3)	C6—H6A	0.9500
O1—C1	1.405 (3)	C7—C8	1.384 (3)
O2—C2	1.360 (3)	C7—C12	1.394 (3)
O2—C15	1.434 (3)	C8—C9	1.394 (3)
N1—C13	1.145 (3)	C8—H8A	0.9500
N2—C14	1.145 (3)	C9—C10	1.408 (3)
C1—C6	1.377 (3)	C9—C13	1.434 (3)
C1—C2	1.393 (3)	C10—C11	1.395 (3)
C2—C3	1.395 (3)	C10—C14	1.441 (3)
C3—C4	1.382 (4)	C11—C12	1.381 (3)
C3—H3A	0.9500	C11—H11A	0.9500
C4—C5	1.379 (4)	C12—H12A	0.9500
C4—H4A	0.9500	C15—H15A	0.9800
C5—C6	1.388 (3)	C15—H15B	0.9800
C5—H5A	0.9500	C15—H15C	0.9800
			
C7—O1—C1	117.45 (17)	C7—C8—C9	118.8 (2)
C2—O2—C15	116.7 (2)	C7—C8—H8A	120.6
C6—C1—C2	122.1 (2)	C9—C8—H8A	120.6
C6—C1—O1	119.1 (2)	C8—C9—C10	120.9 (2)
C2—C1—O1	118.8 (2)	C8—C9—C13	120.1 (2)
O2—C2—C1	116.0 (2)	C10—C9—C13	119.0 (2)
O2—C2—C3	126.0 (2)	C11—C10—C9	119.1 (2)
C1—C2—C3	118.0 (2)	C11—C10—C14	121.5 (2)
C4—C3—C2	119.8 (2)	C9—C10—C14	119.4 (2)
C4—C3—H3A	120.1	C12—C11—C10	120.0 (2)
C2—C3—H3A	120.1	C12—C11—H11A	120.0
C5—C4—C3	121.5 (2)	C10—C11—H11A	120.0
C5—C4—H4A	119.2	C11—C12—C7	120.4 (2)
C3—C4—H4A	119.2	C11—C12—H12A	119.8
C4—C5—C6	119.3 (2)	C7—C12—H12A	119.8
C4—C5—H5A	120.4	N1—C13—C9	179.0 (3)
C6—C5—H5A	120.4	N2—C14—C10	178.6 (2)
C1—C6—C5	119.3 (2)	O2—C15—H15A	109.5
C1—C6—H6A	120.4	O2—C15—H15B	109.5
C5—C6—H6A	120.4	H15A—C15—H15B	109.5
O1—C7—C8	123.6 (2)	O2—C15—H15C	109.5
O1—C7—C12	115.53 (19)	H15A—C15—H15C	109.5
C8—C7—C12	120.9 (2)	H15B—C15—H15C	109.5

### 4-(2-Methoxyphenoxy)benzene-1,2-dicarbonitrile (o-C15H10N2O2) Hydrogen-bond geometry (Å, º)

**Table:**

*D*—H···*A*	*D*—H	H···*A*	*D*···*A*	*D*—H···*A*
C3—H3*A*···O2^i^	0.95	2.95	3.629 (3)	129
C5—H5*A*···N2^ii^	0.95	2.66	3.394 (3)	134
C5—H5*A*···N1^iii^	0.95	2.90	3.557 (3)	128
C5—H5*A*···O1^iv^	0.95	2.96	3.453 (3)	113
C8—H8*A*···N2^v^	0.95	2.69	3.469 (3)	140
C11—H11*A*···O2^vi^	0.95	2.82	3.735 (3)	161
C11—H11*A*···O1^vi^	0.95	2.75	3.546 (3)	142
C12—H12*A*···N1^vii^	0.95	2.44	3.199 (3)	137

Symmetry codes: (i) −*x*+2, *y*+1/2, −*z*+3/2; (ii) −*x*+1/2, −*y*+1, *z*+1/2; (iii) −*x*+1, *y*−1/2, −*z*+3/2; (iv) −*x*+1, *y*+1/2, −*z*+3/2; (v) *x*+1/2, −*y*+3/2, −*z*+1; (vi) *x*−1/2, −*y*+1/2, −*z*+1; (vii) *x*, *y*−1, *z*.

### 4-(3-Methoxyphenoxy)benzene-1,2-dicarbonitrile (m-C15H10N2O2) Crystal data

**Table:**

C_15_H_10_N_2_O_2_	*Z* = 2
*M**_r_* = 250.25	*F*(000) = 260
Triclinic, *P*1	*D*_x_ = 1.371 Mg m^−^^3^
*a* = 8.0609 (3) Å	Mo *K*α radiation, λ = 0.71073 Å
*b* = 8.4672 (4) Å	Cell parameters from 9781 reflections
*c* = 9.9999 (4) Å	θ = 2.7–30.1°
α = 104.638 (1)°	µ = 0.09 mm^−^^1^
β = 95.078 (1)°	*T* = 100 K
γ = 110.570 (1)°	Prism, colorless
*V* = 606.31 (4) Å^3^	0.45 × 0.30 × 0.27 mm

### 4-(3-Methoxyphenoxy)benzene-1,2-dicarbonitrile (m-C15H10N2O2) Data collection

**Table:**

Bruker D8 Quest (CMOS) diffractometer	3040 reflections with *I* > 2σ(*I*)
Radiation source: microfocus tube	*R*_int_ = 0.017
ω scans	θ_max_ = 29.6°, θ_min_ = 2.2°
Absorption correction: multi-scan (SADABS; Krause *et al.*, 2015)	*h* = −11→11
*T*_min_ = 0.903, *T*_max_ = 0.971	*k* = −11→11
10029 measured reflections	*l* = −13→13
3395 independent reflections	

### 4-(3-Methoxyphenoxy)benzene-1,2-dicarbonitrile (m-C15H10N2O2) Refinement

**Table:**

Refinement on *F*^2^	Primary atom site location: dual
Least-squares matrix: full	Secondary atom site location: difference Fourier map
*R*[*F*^2^ > 2σ(*F*^2^)] = 0.039	Hydrogen site location: inferred from neighbouring sites
*wR*(*F*^2^) = 0.105	H-atom parameters constrained
*S* = 1.06	*w* = 1/[σ^2^(*F*_o_^2^) + (0.0528*P*)^2^ + 0.2143*P*] where *P* = (*F*_o_^2^ + 2*F*_c_^2^)/3
3395 reflections	(Δ/σ)_max_ < 0.001
173 parameters	Δρ_max_ = 0.37 e Å^−^^3^
0 restraints	Δρ_min_ = −0.27 e Å^−^^3^

### 4-(3-Methoxyphenoxy)benzene-1,2-dicarbonitrile (m-C15H10N2O2) Special details

**Table:**

Geometry. All esds (except the esd in the dihedral angle between two l.s. planes) are estimated using the full covariance matrix. The cell esds are taken into account individually in the estimation of esds in distances, angles and torsion angles; correlations between esds in cell parameters are only used when they are defined by crystal symmetry. An approximate (isotropic) treatment of cell esds is used for estimating esds involving l.s. planes.

### 4-(3-Methoxyphenoxy)benzene-1,2-dicarbonitrile (m-C15H10N2O2) Fractional atomic coordinates and isotropic or equivalent isotropic displacement parameters (Å^2^)

**Table:**

	*x*	*y*	*z*	*U*_iso_*/*U*_eq_	
O1	0.56496 (10)	0.59440 (10)	0.80203 (7)	0.01795 (16)	
O2	0.87906 (10)	0.47550 (9)	1.17760 (7)	0.01716 (16)	
N1	0.95411 (13)	0.23336 (15)	0.44743 (10)	0.0277 (2)	
N2	0.46049 (13)	−0.06002 (13)	0.21559 (10)	0.0253 (2)	
C1	0.69339 (13)	0.64698 (13)	0.92463 (10)	0.01494 (18)	
C2	0.72490 (12)	0.52173 (12)	0.97939 (9)	0.01389 (18)	
H2A	0.668855	0.398795	0.929816	0.017*	
C3	0.84135 (13)	0.58336 (12)	1.10919 (9)	0.01423 (18)	
C4	0.92411 (14)	0.76450 (13)	1.18106 (10)	0.01779 (19)	
H4A	1.004512	0.805311	1.269112	0.021*	
C5	0.88827 (14)	0.88403 (13)	1.12324 (11)	0.0203 (2)	
H5A	0.943632	1.007075	1.172592	0.024*	
C6	0.77200 (14)	0.82629 (13)	0.99356 (10)	0.0186 (2)	
H6A	0.747571	0.908341	0.953705	0.022*	
C7	0.55405 (13)	0.45928 (12)	0.68822 (9)	0.01418 (18)	
C8	0.70450 (12)	0.42664 (12)	0.65164 (9)	0.01438 (18)	
H8A	0.820847	0.493469	0.709832	0.017*	
C9	0.67994 (12)	0.29370 (12)	0.52775 (9)	0.01387 (18)	
C10	0.50752 (12)	0.19245 (12)	0.44232 (9)	0.01434 (18)	
C11	0.35970 (13)	0.22881 (13)	0.48119 (10)	0.01664 (19)	
H11A	0.242865	0.161880	0.423787	0.020*	
C12	0.38297 (13)	0.36220 (13)	0.60321 (10)	0.01613 (19)	
H12A	0.282369	0.387620	0.629065	0.019*	
C13	0.83370 (13)	0.26098 (14)	0.48459 (10)	0.0185 (2)	
C14	0.48277 (13)	0.05290 (13)	0.31588 (10)	0.01762 (19)	
C15	0.79525 (14)	0.28892 (13)	1.10841 (11)	0.0189 (2)	
H15A	0.829282	0.225820	1.168675	0.028*	
H15B	0.835383	0.260815	1.018882	0.028*	
H15C	0.663676	0.252294	1.090253	0.028*	

### 4-(3-Methoxyphenoxy)benzene-1,2-dicarbonitrile (m-C15H10N2O2) Atomic displacement parameters (Å^2^)

**Table:**

	*U*^11^	*U*^22^	*U*^33^	*U*^12^	*U*^13^	*U*^23^
O1	0.0233 (4)	0.0224 (4)	0.0123 (3)	0.0155 (3)	0.0020 (3)	0.0032 (3)
O2	0.0211 (3)	0.0165 (3)	0.0143 (3)	0.0086 (3)	0.0001 (3)	0.0046 (3)
N1	0.0205 (4)	0.0418 (6)	0.0197 (4)	0.0167 (4)	0.0019 (3)	0.0014 (4)
N2	0.0272 (5)	0.0235 (5)	0.0203 (4)	0.0083 (4)	0.0016 (4)	0.0019 (3)
C1	0.0167 (4)	0.0193 (4)	0.0115 (4)	0.0094 (3)	0.0045 (3)	0.0051 (3)
C2	0.0156 (4)	0.0145 (4)	0.0126 (4)	0.0073 (3)	0.0041 (3)	0.0034 (3)
C3	0.0163 (4)	0.0164 (4)	0.0124 (4)	0.0081 (3)	0.0049 (3)	0.0052 (3)
C4	0.0195 (4)	0.0176 (5)	0.0135 (4)	0.0060 (4)	0.0021 (3)	0.0022 (3)
C5	0.0246 (5)	0.0141 (4)	0.0198 (5)	0.0064 (4)	0.0047 (4)	0.0027 (4)
C6	0.0240 (5)	0.0170 (4)	0.0184 (4)	0.0105 (4)	0.0063 (4)	0.0068 (4)
C7	0.0187 (4)	0.0165 (4)	0.0110 (4)	0.0097 (3)	0.0042 (3)	0.0059 (3)
C8	0.0139 (4)	0.0178 (4)	0.0123 (4)	0.0070 (3)	0.0019 (3)	0.0050 (3)
C9	0.0136 (4)	0.0179 (4)	0.0122 (4)	0.0078 (3)	0.0025 (3)	0.0056 (3)
C10	0.0148 (4)	0.0164 (4)	0.0126 (4)	0.0063 (3)	0.0022 (3)	0.0055 (3)
C11	0.0126 (4)	0.0212 (5)	0.0167 (4)	0.0060 (3)	0.0022 (3)	0.0076 (4)
C12	0.0150 (4)	0.0222 (5)	0.0160 (4)	0.0100 (4)	0.0055 (3)	0.0090 (3)
C13	0.0170 (4)	0.0247 (5)	0.0127 (4)	0.0096 (4)	0.0003 (3)	0.0026 (3)
C14	0.0159 (4)	0.0196 (5)	0.0166 (4)	0.0060 (3)	0.0012 (3)	0.0062 (4)
C15	0.0244 (5)	0.0160 (4)	0.0181 (4)	0.0099 (4)	0.0029 (4)	0.0058 (3)

### 4-(3-Methoxyphenoxy)benzene-1,2-dicarbonitrile (m-C15H10N2O2) Geometric parameters (Å, º)

**Table:**

O1—C7	1.3658 (11)	C6—H6A	0.9500
O1—C1	1.3959 (11)	C7—C12	1.3939 (14)
O2—C3	1.3669 (11)	C7—C8	1.3942 (13)
O2—C15	1.4303 (12)	C8—C9	1.3914 (12)
N1—C13	1.1464 (14)	C8—H8A	0.9500
N2—C14	1.1468 (14)	C9—C10	1.4067 (13)
C1—C6	1.3782 (14)	C9—C13	1.4404 (13)
C1—C2	1.3954 (13)	C10—C11	1.3946 (13)
C2—C3	1.3915 (13)	C10—C14	1.4384 (13)
C2—H2A	0.9500	C11—C12	1.3834 (13)
C3—C4	1.3975 (13)	C11—H11A	0.9500
C4—C5	1.3835 (14)	C12—H12A	0.9500
C4—H4A	0.9500	C15—H15A	0.9800
C5—C6	1.3934 (15)	C15—H15B	0.9800
C5—H5A	0.9500	C15—H15C	0.9800
			
C7—O1—C1	120.56 (7)	C9—C8—C7	118.33 (8)
C3—O2—C15	117.05 (7)	C9—C8—H8A	120.8
C6—C1—C2	122.83 (9)	C7—C8—H8A	120.8
C6—C1—O1	116.08 (8)	C8—C9—C10	121.08 (8)
C2—C1—O1	120.80 (8)	C8—C9—C13	119.63 (8)
C3—C2—C1	117.62 (8)	C10—C9—C13	119.28 (8)
C3—C2—H2A	121.2	C11—C10—C9	119.29 (8)
C1—C2—H2A	121.2	C11—C10—C14	119.99 (8)
O2—C3—C2	123.86 (8)	C9—C10—C14	120.72 (8)
O2—C3—C4	115.26 (8)	C12—C11—C10	120.09 (9)
C2—C3—C4	120.85 (9)	C12—C11—H11A	120.0
C5—C4—C3	119.61 (9)	C10—C11—H11A	120.0
C5—C4—H4A	120.2	C11—C12—C7	119.99 (9)
C3—C4—H4A	120.2	C11—C12—H12A	120.0
C4—C5—C6	120.85 (9)	C7—C12—H12A	120.0
C4—C5—H5A	119.6	N1—C13—C9	178.59 (10)
C6—C5—H5A	119.6	N2—C14—C10	178.88 (11)
C1—C6—C5	118.24 (9)	O2—C15—H15A	109.5
C1—C6—H6A	120.9	O2—C15—H15B	109.5
C5—C6—H6A	120.9	H15A—C15—H15B	109.5
O1—C7—C12	115.57 (8)	O2—C15—H15C	109.5
O1—C7—C8	123.07 (8)	H15A—C15—H15C	109.5
C12—C7—C8	121.20 (9)	H15B—C15—H15C	109.5

### 4-(3-Methoxyphenoxy)benzene-1,2-dicarbonitrile (m-C15H10N2O2) Hydrogen-bond geometry (Å, º)

**Table:**

*D*—H···*A*	*D*—H	H···*A*	*D*···*A*	*D*—H···*A*
C2—H2*A*···N2^i^	0.95	2.64	3.5839 (13)	175
C8—H8*A*···O2^ii^	0.95	2.47	3.3447 (11)	153
C11—H11*A*···N1^iii^	0.95	2.62	3.2718 (13)	126
C12—H12*A*···N1^iii^	0.95	2.74	3.3325 (13)	121
C12—H12*A*···O2^iv^	0.95	2.67	3.5343 (12)	151

Symmetry codes: (i) −*x*+1, −*y*, −*z*+1; (ii) −*x*+2, −*y*+1, −*z*+2; (iii) *x*−1, *y*, *z*; (iv) −*x*+1, −*y*+1, −*z*+2.
